# Human tissue factor pathway inhibitor-2 suppresses the wound-healing activities of human Tenon’s capsule fibroblasts in vitro

**Published:** 2009-11-12

**Authors:** Yuan Jing, Yu Jian-Xiong

**Affiliations:** 1 Department of Ophthalmology, Renmin Hospital of Wuhan University, Wuhan, China; 2 Department of Gastrointestinal Surgery, Renmin Hospital of Wuhan University, Wuhan, China

## Abstract

**Purpose:**

Human tissue Factor Pathway Inhibitor-2 (TFPI-2) is a potent inhibitor of plasmin, which activates metalloproteinases involved in extracellular matrix degradation. Its secretion in the extracellular matrix makes TFPI-2 a potential inhibitor of tumor cell invasion. However, no studies have yet evaluated the wound-healing activities of human Tenon’s capsule fibroblasts (hTCFs). The aim of the study is to elucidate the effect of TFPI-2 overexpression on hTCF proliferation and migration, to determine whether TFPI-2 may act as an antiscarring agent in vivo after glaucoma filtration surgery.

**Methods:**

Plasmid vector pBos-Cite-neo/TFPI-2 was transfected into hTCFs with Lipofectamine 2000. After selection by G418, transfected, non-transfected, and mock-transfected cells were screened for TFPI-2 mRNA and protein by reverse transcription-PCR and western blot analysis respectively. Cell proliferation and viability were determined by 3-(4,5-Dimethylthiazol-2-yl)-2,5-diphenyltetrazolium bromide (MTT) assay and flow cytometry. Cell migration was studied on restrained collagen gels and with a scratch-wound assay.

**Results:**

TFPI-2 expression of mRNA and protein was confirmed in transfected cells. The transfected, non-transfected, and mock-transfected cells showed no significant difference in cell proliferation and apoptosis, with TFPI-2 found not to be cytotoxic in hTCFs. Overexpression of TFPI-2 significantly suppressed cell migration three- to four-fold on collagen gel for 2 weeks and in the scratch-wound assay for 2 d (39.27±2.40% versus 16.43±1.10% at 1 d, and 79.0±3.04% versus 30.13±2.1% at 2 d).

**Conclusions:**

TFPI-2 expression may strongly inhibit the migration ability of hTCFs in vitro, making it a promising candidate for novel therapies to minimize scar development after glaucoma drainage surgery.

## Introduction

Trabeculectomy is the most frequently used surgical method to reduce intraocular pressure in patients with glaucoma unresponsive to medical therapy. However, excessive scarring of the filtering bleb after glaucoma filtration surgery can lead to an increase in intraocular pressure, and is the most important cause of treatment failure. A variety of antimetabolites, such as 5-fluorouracil and mitomycin C, has been shown to be clinically effective at preventing bleb failure after filtration surgery [1,2]. Their antifibrotic effect has been shown to derive mostly from the inhibition of human Tenon’s capsule fibroblasts (hTCFs) proliferation, as well as from apoptotic cell death [3]. However, these agents are associated with significant adverse side effects and postoperative complications, such as ocular hypotony, following choroidal detachment and hypotonic maculopathy, progressive thinning of the filtering bleb following bleb infection, and endophthalmitis [4,5]. Most studies on the filtering bleb healing process and its modulation have concentrated on fibroblast proliferation. However, in some high-risk patients, even after antiproliferative treatment, surgery still fails, in part due to residual activity of the growth-arrested cells and their interaction with surrounding untreated fibroblasts. Therefore, alternative targets to prevent scar formation after trabeculectomy are needed.

The wound-healing processes include proliferation, migration, synthesis of extracellular matrix (ECM) components, and collagen contraction by hTCFs (the key cells involved in the subconjunctival wound-healing response). After injury, quiescent fibroblasts in the surrounding matrix are activated; they proliferate, and migrate into the wound site to deposit and remodel a new matrix, resulting in tissue fibrosis and scar formation. This process involves a family of enzymes capable of cleaving components of the ECM, such as matrix metalloproteinases (MMPs). Human tissue factor pathway inhibitor-2 (TFPI-2), an inhibitor of MMPs, may inhibit scarring after glaucoma filtration surgery. TFPI-2 is a member of the same Kunitz-type serine protease inhibitor family as tissue factor pathway inhibitor-1 (TFP-1). TFPI-2 contains three Kunitz domains arranged in tandem with a high degree of conservation. The basic carboxy terminus of TFPI-2 mediates ionic interactions that associate this protein with glycosaminoglycans in ECMs [6,7]. Studies have demonstrated that TFPI-2 is a strong serine protease inhibitor with broad inhibitory spectra, whose expression can decrease the invasion capacity of various tumor cells [8-12]. Its effect on the wound-healing activities of hTCFs is unknown. Therefore, the aim of the present study was to elucidate the effect of TFPI-2 on hTCF proliferation and migration to determine its suitability as an antiscarring agent for in vivo use after glaucoma filtration surgery.

## Methods

### Cell culture

Cultures of human Tenon's capsule were established from patients undergoing routine cataract surgery. The tenets of the Declaration of Helsinki were followed in the collection of human material, and patients’ consent was obtained. Excised specimens were dissected into 1-2 mm cubes and maintained in Dulbecco's modified Eagle's medium (Gibco BRL, Gaithersburg, MD), supplemented with 20 mM HEPES, 10% fetal bovine serum (FCS), penicillin G (100 IU/ml), streptomycin (100 μg/ml), and amphotericin B (0.25 mg/ml), then cultured in an incubator at 37 °C in 5% carbon dioxide and 95% humidified air. The hTCFs migrating from these tissues were harvested and subcultured using 0.05% trypsin and 0.02% EDTA (Gibco BRL, Gaithersburg, MD) after approximately 2 weeks. Cells cultured for 4-5 passes were used in this study. All experiments were repeated more than twice.

### Preparation and transformations of the human tissue factor pathway inhibitor-2 expression vector

The TFPI-2 expression vector (pBos-Cite-neo/TFPI-2) was kindly donated by Dr. Zhong Ren (Department of Hematology, Union Hospital, Wuhan, China). The plasmids were extracted by being dissolved in alkali, and then purified using a precipitate of lithium chloride (LiCl) and polyethylene glycol (PEG). Nucleic acid purity was estimated by light absorbance of the 260 nm and 280 nm wavelengths. If the ratio of A260/A280 was between 1.8 and 2.0, the DNA had high purity. Then, plasmids were adjusted to 1.0 g/l and preserved at 4 ºC. The TFPI-2 expression vector or the empty pBos-Cite-neo vector was transfected into hTCFs with Lipofectamine reagent (Gibco BRL, Gaithersburg, MD) according to the manufacturer’s protocol. Transfected cells were selected and cultured in medium containing 500 mg/ml of G418 (Gibco BRL, Gaithersburg, MD) for 2-3 weeks. The colonized cells were isolated, amplified, and used in subsequent experiments.

### Reverse transcription-PCR and western blot analysis

Total RNA was isolated with TRIzol Reagent (Invitrogen, Carlsbad, CA). Reverse-transcription PCR (RT-PCR) was carried out using the one-step RT-PCR system (Promega, Madison, WI). The TFPI-2 primers were 5'-GTC GAT TCT GCT GCT TTT CC-3' sense primer, corresponding to nucleotides 64-84 of the published sequence [13] and 5'-ATG GAA TTT TCT TTG GTG CG-3' antisense primer (nucleotide 484-504). Thirty-five cycles were performed, at 94 ºC for 1 min, then at 60 ºC for 1 min, then at 72 ºC for 1 min. β-Actin was amplified as an internal control with primer 5'-GAA ACT ACC TTC AAC TCC ATC-3' and antisense primer 5'-CGA GGC CAG GAT GGA GCC GCC-3', with predicted products of 440 bp and 219 bp, respectively. Each reaction (5 μl) was separated on 1.5% agarose gel, and visualized by ethidium bromide staining and photography. Western blotting was carried out as described previously [10]. Proteins were visualized with HRP-conjugated anti-rabbit immunoglobulin G (1:5000; Santa Cruz Biotechnology, Inc., Santa Cruz, CA), followed by western blotting (Pierce Biotechnology, Rockford, IL) and photography.

### Cell proliferation and analysis of apoptosis assay

3-(4,5-Dimethylthiazol-2-yl)-2,5-diphenyltetrazolium bromide (MTT) cell viability assay (CellTiter 96 Aqueous One Solution Cell Proliferation Assay; Promega, Madison, WI) was used to evaluate cell proliferation following the manufacturer's instructions. Briefly, TFPI-2-transfected cells (F-TFPI-2), mock-transfected cells (F-V), and non-transfected cells (F-P; all at 1×10^4^ cells/ml) were seeded into 96-well plates and cultivated for 14 d. After days 2, 4, 6, 8, 10, 12, and 14, a 20 μl MTT stock solution (5 mg/ml) was added to each well, and the cells were incubated at 37 °C for 3 h. The solution was discarded by gently inverting the plates, and the wells were filled with 200 μl of lysis solution (0.6% acetic acid and 10% sodium dodecyl sulfate in dimethyl sulfoxide). After the plates were shaken vigorously for 20 min, absorbances in each well were read with a spectrophotometric plate reader at 570 nm.

To quantitate apoptosis, cells from F-P, F-V, and F-TFPI-2 were harvested, washed with cold PBS twice and centrifuged 3X at 12,000 rpm for 5 min each at 4 ºC, The staining of apoptotic cells was performed as follows: cells were resuspended in 1X binding buffer, with cell concentration adjusted to 1×10^6^/ml. A cell suspension if 100 μl was added to a tube, followed by 5 μl FITC-Annexin V (Jingmei Biotech, Shenzhen, China) and 10 μl PI. The mixture was incubated for 15 min in the dark at room temperature, and 400 μl 1× binding buffer was added. Flow cytometric measurements were performed in a FACS Vantage sorter (Becton Dickinson, Heidelberg, Germany) and analyzed using CelQuest software (Becton Dickinson, Heidelberg, Germany). The percentage of apoptotic cells was measured and the data shown as the mean±SD of the results of three independent experiments from each group.

### Cell migration from spheroids

To evaluate hTCFs with or without TFPI-2 transfection migration, collagen gels were prepared by mixing a cold type I collagen solution with 10× DMEM, 0.2 M HEPES, and 0.2 N NaOH on ice at a ratio of 8:1:1 (vol/vol/vol). Of this solution, 25 μl were cast on a sterile slide placed in a 100 mm Petri dish. The gels were kept moist by the addition of 1.0 ml of culture medium without serum, and allowed to polymerize at 37 °C. Cells of F-P and F-TFPI-2 were harvested by trypsinization, resuspended in a culture medium without serum at a density of 1×10^6^ cells/ml, seeded onto the polymerized gels, and allowed to form cell aggregates for 2-3 h at 37 °C. The gels were then covered with culture medium containing 10% FCS. The aggregate was incubated, and the cells were allowed to migrate onto the collagen gel for 14 d. Immediately after cell attachment (T0) and at the time points specified above for the 14 d incubation, images of cell migration from the initial aggregates to the monolayers were obtained using an inverted microscope (Olympus 1×71, Olympus Corporation, Tokyo, Japan) attached to a camera (Olympus DP12). At day 14, the cells were fixed with methanol for 10 min and processed for immunofluorescence staining with Hoechst33342 (Hoechst Celanese, Frankfurt, Germany).

### Scratch-wound assay

F-TFPI-2 cells treated with or without anti-TFPI-2 antibodies (Santa Cruz Biotechnology Inc., Santa Cruz, CA) were harvested by trypsinization and seeded in 24-well plates at a concentration of 5,000 cells/well, and then grown until reaching confluence. F-P cells were seeded into cultured plates as a control. A linear wound was gently introduced in the center of the cell monolayers using sterile 1.0 mm wide silicon strips, followed by extensive washing with the growth medium to remove the cellular debris. Then, the cells were incubated and wound closure monitored for 48 h. Cell images of marked regions along the wound area were obtained using an inverted microscope (Olympus 1×71) attached to a camera (Olympus DP12) immediately after creating the wound and at 48 h.

### Statistical analysis

Values are reported as mean±SD or as median and range. Each experiment was repeated at least three times. Statistical significance of the differences between experimental groups was determined by Student’s t test. Differences were considered significant when p<0.05. All statistical analyses were conducted with SPSS version 16.0 (SPSS, Inc., Chicago, IL).

## Results

### Identification and expression of tissue factor pathway inhibitor-2 after gene transfection

**Figure 1:**
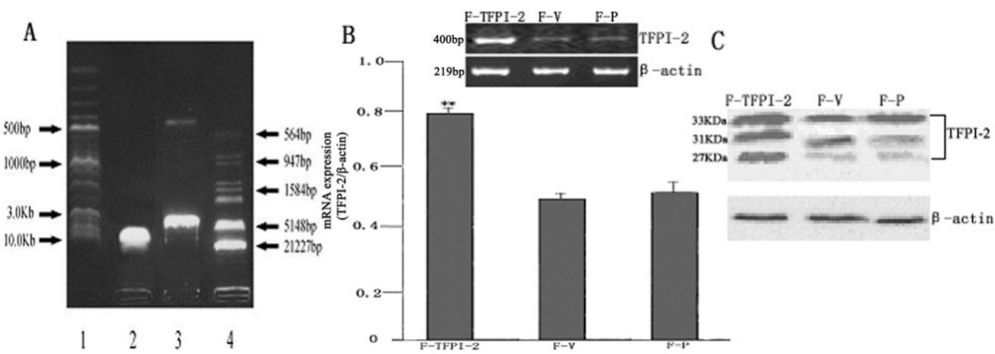
Identification and expression of tissue factor pathway inhibitor after gene transfection. **A**: Restriciton enzyme analysis of TFPI-2 plasmids digested with XbaI and KpnI. Line 1 is the 2-Log Marker. Line 2 is the pBos-Cite-neo/TFPI-2. Line 3 is the pBos-Cite-neo/TFPI-2 digested with XbaI and KpnI. Line 4 is the λDNA/EcoRI+HindIII Markers. **B**: Semi-quantification of TFPI-2 mRNA expression in hTCFs after transfection with an empty vector or TFPI-2 expression vector was assessed by RT-PCR. RT-PCR for β-actin mRNA was performed as a control. Relative OD Value (OD TFPI-2/OD β-actin) was used to evaluate the expression level of TFPI-2. Data are presented as mean value±SD, the double asterisk indicates a p<0.001, Student’s t-test. **C**: Western blot analysis for the expression of TFPI-2 in hTCFs after transfection with an empty vector or TFPI-2 expression vector. Each protein fraction was subjected to sodium dodecyl sulfate-polyacrylamide gel electrophoresis on 15% acrylamide gels, transferred onto nitrocellulose membranes, and probed with a rabbit anti-human TFPI-2 antibody. Molecular sizes of the detected bands are shown to be 33, 31, and 27 kDa.

After pBos-Cite-neo/TFPI-2 was digested by XbaI and KpnI, two fragments of 5.05 kb and 440 bp were seen in 1% agarose gel electrophoresis, indicating 440 base pairs between the polyclonal sites XbaI and KpnI (Figure 1A), confirming that the plasmid of pBos-Cite-neo/ TFPI-2 contained the human TFPI-2 gene. The ratio of OD260/OD280 was 1.80, indicating plasmid purity.

After 48 h, hTCFs were derived from areas around the tissue. On days 4-5, cells grown around the tissue were fusiform. At 2 weeks, cells were reticular. Subcultured cells were uniformly fusiform. Following transfection with Lipofectamine 2000, cells were selected by G418, and stable transfectants showed the same shape as non-transfected cells. Both the mRNA and protein expression of TFPI-2 were confirmed after gene transfection by RT-PCR and western blot, respectively. TFPI-2 mRNA was expressed strongly in F-TFPI-2, but only slightly in F-V and F-P, suggesting that hTCFs strongly expressed TFPI-2 mRNA after being transfected with TFPI-2 (Figure 1B). Consistent with RT-PCR results, three apparent bands with molecular masses of 33, 31, and 27 kDa were detected in the ECM of F-TFPI-2, but were only weakly detected in the ECMs of F-V and F-P cells, indicating strong, stable TFPI-2 protein expression in the ECM of F-TFPI-2, but only slight expression in the ECMs of F-V and F-P (Figure 1C).

### Effect of tissue factor pathway inhibitor-2 on cell proliferation and apoptosis

**Figure 2:**
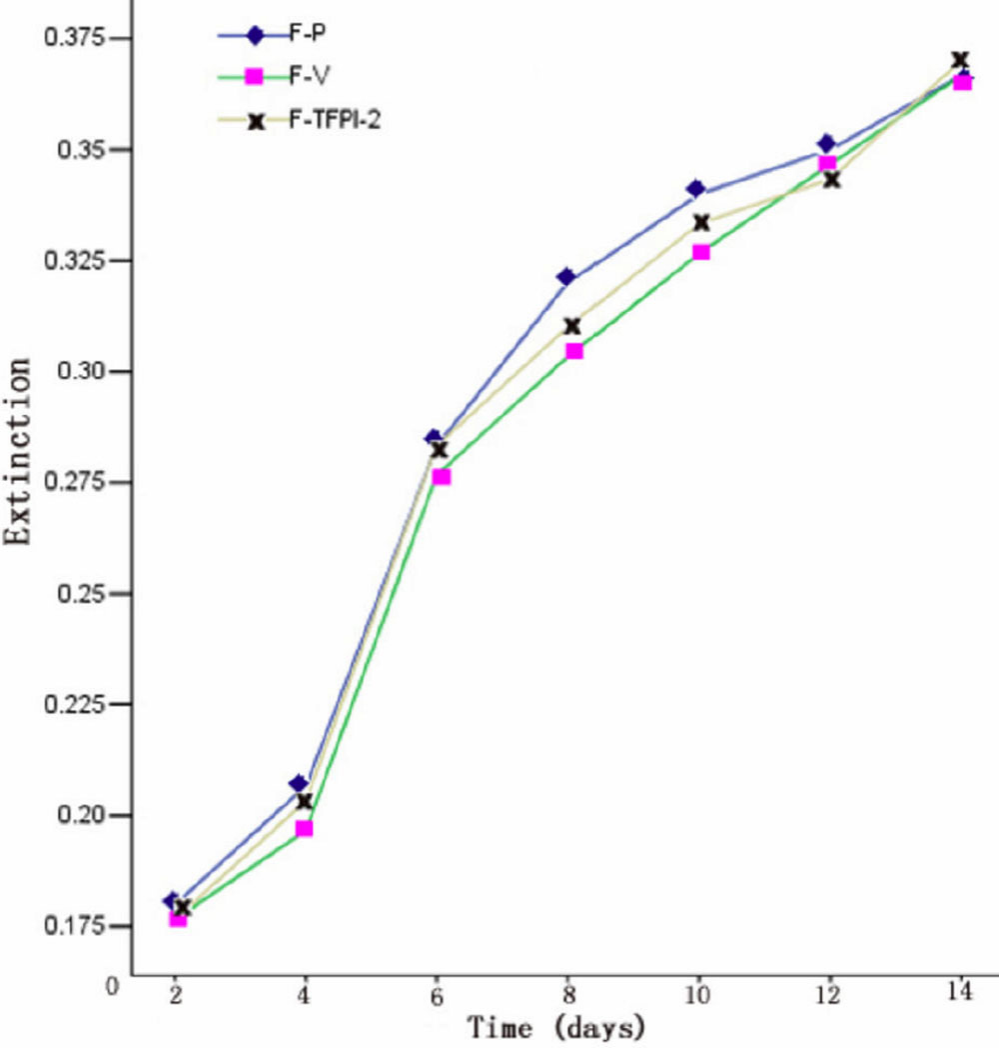
MTT cell viability assay of F-P, F-V, and F-TFPI-2. Extinctions of hTCFs were not significantly different among the three groups, showing that either mock transfected cells or TFPI-2 transfected cells did not significantly influence metabolic cell proliferation activity.

MTT cell viability assay allows evaluation of cell metabolic activity such as cell number, cell metabolism, and mitochondrial activation. As seen in Figure 2, the absorbance of the three groups did not differ significantly, indicating that neither the vector construct itself nor TFPI-2 gene transfection significantly influenced cell proliferation.

**Figure 3:**
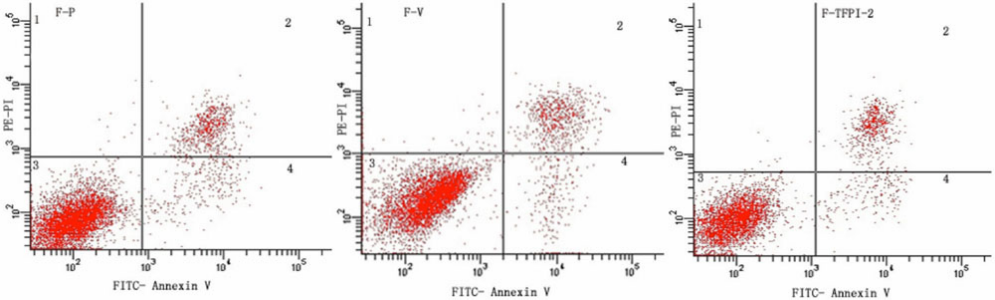
Contour diagram of FITC-Annexin V/PI flow cytometry of hTCFs for F-P, F-V, and F-TFPI-2. The lower right quadrant (region 4) represents the apoptotic cells. The percentages of the apoptotic cells population were 4.0%, 4.2%, and 4.3%, respectively. One representative experiment out of three was shown.

By flow cytometry, we evaluated cell apoptosis. With FITC-Annexin V/PI double staining, normal live cells were represented as FITC-negative and PI-positive, early apoptotic cells as FITC-positive and PI-negative, and necrotic cells as FITC-positive and PI-positive. The percentage of apoptotic cell populations in F-P, F-V, and F-TFPI-2 were, respectively, 4.09±0.09%, 4.14±0.06%, and 4.22±0.07% (again respectively, p=0.129, F-P versus F-TFPI-2; p=0.196, F-V versus F-TFPI-2; and p=0.509, F-P versus F-V). This indicated no significant effect on cell apoptosis of either mock-transfected cells or TFPI-2-transfected cells. Representative experiments are shown in Figure 3.

### Human tissue factor pathway inhibitor-2 inhibits human Tenon’s capsule fibroblasts migration in vitro

**Figure 4:**
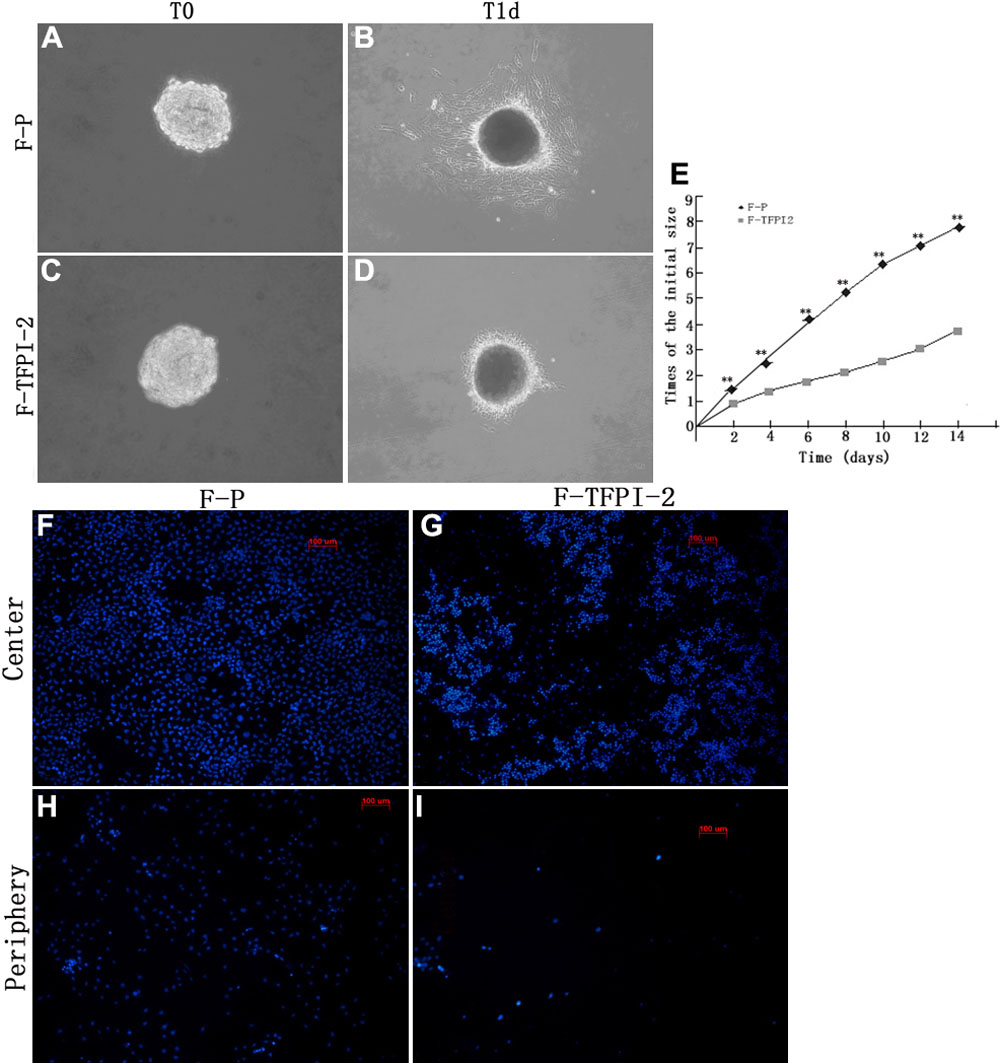
Effect of TFPI-2 on the migration of hTCFs on collagen gel. **A-D**: Cells from F-P and F-TFPI-2 were cultured on a restrained collagen gels in the initial spheroids. They were allowed to migrate for 1 day, and then were viewed under a microscope. **E**: An equal amount of F-P and F-TFPI-2 cells were inoculated on the collagen gels and the changes in the areas occupied by cells were monitored for 14 d. The multiple of the initial area by the migration of cells from the center of the spheroids to the monolayers was measured using a microscope calibrated with a stage and ocular micrometer. The data shown are the mean value±SD of the results of three independent experiments from each group. The double asterisk indicates a p value of <0.001, for the Student’s t-test. **F**-**I**: Representative images of at the center and periphery of the collagen gels are of the migration of F-P and F-TFPI-2 after 14 d.

To determine whether TFPI-2-mediated cells influence migration, we observed the migration of sphere cells on restrained collagen gels. As seen in Figure 4A,B, F-P, cells arranged in initial spheroids spread out on the gel radially at 1 d, migrating to 6-8 times the initial size at 14 d. The confocal micrographs showing blue nuclei staining with Hoechst33342 were equally densely arranged, both at the inoculation zone (in the center) and along the tracks leading to the farthest points reached by the cells (at the periphery) at 14 d (Figure 4F,G). By contrast, F-TFPI-2 cell migration was much lower. These cells had a less polarized morphology, had no significant protruding edge, appeared to be restricted to the inside of the gels (Figure 4C,D), and had reached only twice their initial size at 14 d. Immunostaining of nuclei showed a more clonal arrangement than F-P at the center and few cells at the periphery at 14 d. The area occupied by cells was evaluated to quantify the difference in cell migration between F-P and F-TFPI-2 at 14 d (Figure 4D).

**Figure 5:**
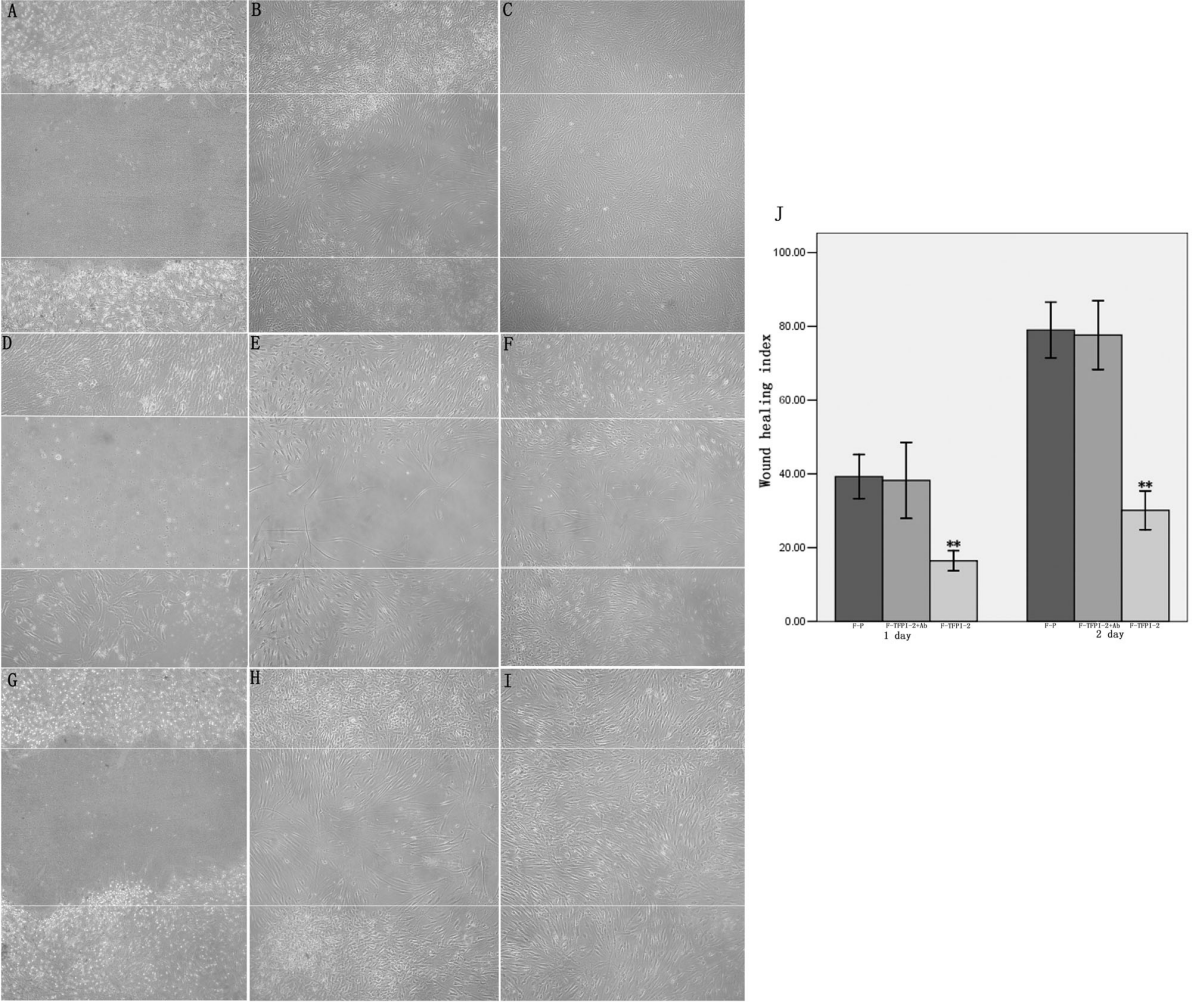
Overexpressioin of TFPI-2 reduces hTCF migration in the scratch-wound assay. **A**-**I**: Phase contrast images of migration of F-P and F-TFPI-2 treated with or without anti-TFPI-2 antibodies wounded with 1 mm strips at day T0, T1, and T2. Bars, 200 μm. **J**: Quantification of cell migration at day T1 and T2. Mean values from 3 different experiments are shown for F-P, F-TFPI-2 with or without anti-TFPI-2 Ab in culture medium. The double asterisk denotes a significant difference from F-P and F-TFPI-2 treated with anti-TFPI-2 antibodies versus F-TFPI-2 at p<0.001.

In the scratch-wound assay, F-P cells and F-TFPI-2 cells treated with anti-TFPI-2 Ab migrated into the wound area and organized a dense cellular network, resulting in nearly complete wound recovery after 2 d (Figure 5A-C,G-I). However, migration of F-TFPI-2 cells into the wound area was significantly suppressed by excess TFPI-2 (Figure 5D-F). Wound coverage, expressed as the percentage of the initial area covered, represented the index of wound healing. Three experiments for F-P, F-TFPI-2 treated with anti-TFPI-2 Ab, and F-TFPI-2 showed wound coverage, respectively, of 39.27±2.40%, 38.23±4.15%, and 16.43±1.10% on day 1, and of 79.00±3.04%, 77.63±3.75%, and 30.13±2.11% at 48 h (Figure 5J).

## Discussion

Most approaches to modifying the wound-healing response after filtering surgery by gene transfer have concentrated on hTCF proliferation. Recent studies show that hTCFs in the focal area remain capable of migration and may contribute to wound scarring even after antiproliferative treatment [2], resulting in the failure of glaucoma filtration surgery. During the scarring response, migration of active hTCFs involves enhanced proteolysis and the means to prevent excessive ECM degradation, which would ultimately serve to inhibit migration. TFPI-2, a 32 kDa serine proteinase inhibitor, is known to play a regulatory role in several processes relevant to the pathogenesis of tumor invasion [6-12]. In vitro, TFPI-2 inhibits extracellular proteolytic activity and TF-mediated coagulation. It may also influence cell migration and proliferation. However, we found no reports of the effect of TFPI-2 on wound-healing in hTCFs. Hence, the present data establishes the endogenous overexpression of TFPI-2, and for the first time, shows by gene transfer that TFPI-2 inhibits the temporal and spatial migration of hTCFs.

This study is the first to investigate the expression of TFPI-2 after successful transfection of pBos-Cite-neo/TFPI-2 into cultured hTCFs. The results show that TFPI-2 mRNA and protein were only slightly expressed in F-V and F-P, but obviously expressed in F-TFPI-2 according to RT-PCR and western blot analyses. This suggested that hTCFs can synthesize and secrete only an extraordinary small quantity of TFPI-2,

, at levels insufficient to protect the integrity of ECMs and block hTCFs in wound scarring. However, it may have been significantly upregulated by gene transfection. Meanwhile, we observed cellular shape and proliferation after transfection to have been unchanged from non-transfected cells, indicating that increased levels of TFPI-2 probably resulted from increased expression of TFPI-2 per hTCF, rather than from the presence of more cells. Our result was different from that of Shinoda [14], who reported TFPI-2 could be expressed in endothelial cells and act as a mitogen for vascular smooth muscle cells, indicating that the cell growth-stimulation of TFPI-2 may be cell type-dependent. In addition, we found no effect of TFPI-2 on hTCF apoptosis, unlike Tasiou [15], who found a pro-apoptotic effect of TFPI-2 in malignant human SNB19 gliomas after transfection with sense TFPI-2 DNA. Kempaiah et al. [10] also found a pro-apoptosis function of TFPI-2, after recombinant TFPI-2 protein was offered to HT-1080 cells by activated caspase-mediated signaling pathways. Because no such role was observed in the present study, we assume the reason to be low expression in normal cells, such as hTCFs, of the receptor to tumor necrosis factor-related apoptosis-inducing ligand (TRAIL). As measured by normal cell proliferation and viability, TFPI-2 was found not to be cytotoxic for hTCFs.

We evaluated the migration of hTCFs on collagen gel and into a wound area created in a cell monolayer. The free-floating fibroblast-populated collagen model in vitro is thought to represent the matrix contraction exerted by the tractional forces generated by cells in vivo as they migrate through the matrix. The in vitro wound-healing model of scraping cells and monitoring for migration to the edge of the wound may be considered a prescreening model, to be used to select for substances to treat wound healing in vivo. The result showed that the administration of TFPI-2 significantly suppresses hTCF migration onto the restrained collagen matrix, as well as into a wound area, as shown by the absence of a distinct leading or trailing edge. Excluding the contribution of cell proliferation from the outcomes, we considered the moderately increased levels of TFPI-2 signaling to have indeed slowed the wound-healing process, by reducing the number of hTCFs arriving on the collagen gel or at the wound site. The upregulation of TFPI-2 as a potent serine proteinase inhibitor may serve to limit proteolysis. This involves the plasmin, trypsin, and matrix metalloproteinases (MMPs) that are upregulated during wound healing, thereby preventing the destruction of the ECMs required for migration. This is a surprising and important finding in the context of antiscarring therapy, because cell migration around wounds significantly contributes to the formation of scar tissue. Its ability to inhibit cell motility may render TFPI-2 a promising candidate for novel therapies for preventing or reducing unwanted wound-healing scars after glaucoma drainage surgery.

In summary, we evaluated the effect of endogenous TFPI-2 of hTCFs on cells’ proliferation and migration in vitro, showing that TFPI-2 significantly inhibits cell migration without inducing apoptosis. This makes it a promising drug for regulating wound healing following glaucoma surgery. Further studies are needed to investigate the gene transduction strategies of TFPI-2 and its activity in vivo.
